# Supplementary material for: Transcriptional repression of NFKBIA triggers constitutive IKK‐ and proteasome‐independent p65/RelA activation in senescence
Source: EMBO J. 2021 Jan 18;40(6):e104296. doi: 10.15252/embj.2019104296 (PMC7957429; doi:10.15252/embj.2019104296)
Supplement: Supplementary file 1 — Appendix [file EMBJ-40-e104296-s009.pdf]

APPENDIX

Table of contents:

pp. 2 - 10 Supplemental methods, primer list

pp. 10 - 16 Supplemental Figure Legends

p. 17 Supplemental References

SUPPLEMENTAL MATERIALS AND METHODS

Cell culture: U2-OS, TIG3, and TIG3 ER+H RAS V12 cells were cultured in DMEM (Gibco) supplemented with 10% FCS (Gibco) and 1x Penicillin/streptomycin (Gibco). Early passage BJ and WI38 cells were obtained from ATCC (Manassas, USA) and were cultured with MEM (Gibco) supplemented with 10% FCS and 1x Penicillin streptomycin. Irradiation was carried out with STS OB 29 with a ^{137}Cs source.

Primary mouse tissue isolation: In brief, kidney tissue was isolated from mice with ubiquitously suppressed NF-κB activity *129;129P2-ctnnb1*$^{tm(NFKBIA\Delta N)1Rsu}$ (∆*N*) and control littermates using collagenase A and dispase II (Roche applied bioscience) according to the manufacturer's protocol. Cells were washed with 1X PBS five times and resuspended in DMEM. Cells were cultured in DMEM and irradiated with 40 Gy.

Immunohistochemistry and immunofluorescence on mouse tissues was performed as described previously (Rehm et al, 2014).

BrdU incorporation: To quantify cell proliferation, the analogue 5-bromo-2'-deoxyuridine (BrdU, BD Biosciences) was added in cell cultures for 12 hours prior to measurement at a concentration of 10 µM. BrdU incorporation is detected by flow cytometry with a FITC-conjugated anti-BrdU antibody. The exact procedure was carried out according to the manufacturer's BrdU Flow kit protocol (BD Biosciences).

cDNA synthesis and quantitative RT-PCR (RT-qPCR): cDNA was produced using iScript cDNA synthesis kit (Bio-Rad) according to manufacturer's protocol. RT-qPCR was run using the CFX96 Real Time System (Bio-Rad) and GoTaq® qPCR Master Mix (Promega). A minimum of two reference genes was used. The normalized expression (ΔΔCq) was calculated by the CFX manager software (Bio-Rad). RT-qPCR results are shown as mean, with the error bar representing the SD.

Mouse RT-qPCR primers

Gene name	Accession no.	Sequence (5'->3')
Sdha	NM_023281	GGAAGCACACCCTCTCATAT
		CTGTAGCTGTCAGGTTTCATC
Hprt1	NM_013556	GGATATGCCCTTGACTATAATGAG
		GGCAACATCAACAGGACTC
Nfkbia	NM_010907.2	AAAATCTCCAGATGCTACCCG
		TCCACTTATAATGTCAGACGCTG
Il6	NM_031168.2	ACAAAGCCAGAGTCCTTCAGAGA
		AGCCACTCCTTCTGTGACTCC
C2	NM_013484.2	CCTGGGCATGGAGACCTCTG
		CACCCATGTTGGACTTTCCGTC
Ccl20	NM_016960.2	CCCAGCACTGAGTACATCAAC
		GTATGTACGAGAGGCAACAGTC
Cxcl12	NM_021704.3	TCTTCGAGAGCCACATCGCC
		AGCCGTGCAACAATCTGAAGG
Icam1	NM_010493.3	GCAGAGGACCTTAACAGTCTAC
		TGGGCTTCACACTTCACAG
Mmp3	NM_010809.2	TAAAGACAGGCACTTTTGGCG
		GTGCCCTCGTATAGCCCAGA
Tnf	NM_013693.3	AACTTCGGGGTGATCGGTCC
		GTGGTTTGCTACGACGTGGG

Human RT-qPCR primers

Gene name	Accession no.	Sequence (5'->3')
RELA	NM_001145138.1<br>NM_001243984.1<br>NM_001243985.1<br>NM_021975.3	CCTGTCCTTTCTCATCCCATC
		ACCTCAATGTCCTCTTTCTGC
IRF1	NM_002198.3<br>NM_001354924.1<br>NM_001354925.1	TGCGAGTGTACCGGATGCTT
		CCCACATGACTTCCTCTTGGC
TNFAIP3	NM_001270507.1<br>NM_001270508.2<br>NM_006290.4	TCCTGCCTTGACCAGGACTTG
		CATTGTGCTCTCCAACACCTCT
NFKBIA	NM_020529.2	GAGGACGAGCTGCCCTATGA
		AGCCCCTTTGCGCTCATAAC
IER3	NM_003897.4	GACCTGACCTGTCTCCGTTTT
		TTTTGGCTGGGTTCGGTTCC
NUAK2	NM_030952.3	ACCACCCTCACATCATTGCCA
		CCGGCTGGCATACTCCATGA
JUNB	NM_002229.2	GGTGGCGGCAGCTACTTTTC
		TGTTGGGGACAATCAGGCGT
CXCL8	NM_000584.4<br>NM_001354840.3	AGCCTTCCTGATTTCTGCAG
		GTCCACTCTCAATCACTCTCAG
CXCL2	NM_002089.3	CTCAACCCCGCATCGCC
		TCCTTCAGGAACAGCCACCAAT
CSF2	NM_000758.3	GGCGTCTCCTGAACCTGAGT
		GTCGGCTCCTGGAGGTCAAA
CXCL3	NM_002090.3	TTTCATCAAACATAGCTCAGTCCTG
		CCAGTTCCCCACCCTGTCA
CXCL5	NM_002994.5	ACCCAGGGAAGACAAGAAGGA
		AACACTTCATTAGCTGAGCTGAAAG
TNF	NM_000594.3	CTCTAATCAGCCCTCTGGCCC
		CAGCTTGAGGGTTTGCTACAACA
CCL4	NM_002984.4	CTCCCAGCCAGCTGTGGTATTC
		CCAGGATTCACTGGGATCAGCA
CXCL10	NM_001565.3	CCAGAATCGAAGGCCATCAAGA
		TCGATTTTGCTCCCCTCTGGT
BIRC3	NM_001165.5<br>NM_182962.3	GTGTTAGACTTACTCAATGCAGAAG
		CCAGGATTGGAATTACACAAGTC
IL1B	NM_000576.2	CCTGAGCTCGCCAGTGAAAT
		GGTCCTGGAAGGAGCACTTCAT
CCL7	NM_006273.4	TGTATATGTCATCTCAGTGCTGTAAA
		GCTTCCATAGGGACATCATATCTTAA

F3	NM_001993.4<br>NM_001178096.2	GACATGGAGACCCCTGCCTG<br>TTTGTAGTGCCTGAAGCGCC
MMP3	NM_002422.3	TGTCCCTCCAGAACCTGGGA<br>TTTGCGCCAAAAGTGCCTGT
MMP1	NM_001145938.2<br>NM_002421.4	TCAGGGGAGATCATCGGGACA<br>CCCTCCAATACCTGGGCCTG
IL6	NM_000600.3<br>NM_001318095.2<br>NM_001371096.1	CCAGTTCCTGCAGAAAAAGGCAA<br>GCAGGCTGGCATTTGTGGTT
CCL20	NM_004591.2<br>NM_001130046.2	CATCAATGCTATCATCTTTCACACA<br>CAAGTGAAACCTCCAACCCCA
C3	NM_000064.3	ATGGGCCAGTGGAAGATCCG<br>TCGAAACTGGGCAGCACGTA
CXCL1	NM_001511.3	AAAGCTTGCCTCAATCCTGC<br>TCAGGAACAGCCACCAGTGAG
IL1A	NM_000575.3<br>NM_001371554.1	TGCTGCTGAAGGAGATGCCTG<br>TGCCGTGAGTTTCCCAGAAGA
CXCL11	NM_005409.5<br>NM_001302123.2	CCTTCCAAGAAGAGCAGCAAAG<br>CCTCTTTTGAACATGGGGAAGC
MMP10	NM_002425.2	TCATTCACAGAGCTCGCCCA<br>GGGAGGTCCGTAGAGAGACTGA
NRG1	NM_013956.5<br>(23 transcript variants)	GTTTACTGGTGATCGCTGCCA<br>TCCGCTGTTTCTTGGTTTTGC
CFB	NM_001710.5	GGTTACACTCTCCGGGGCTC<br>CAGTACCCCGCTCCGTTGTC
NFKBIZ	NM_031419.4<br>NM_001005474.2	AAGGATGCAGATGGTGACAC<br>CAAGAACATAGGAAAGTGCCC
ICAM1	NM_000201.2	CCATCTACAGCTTTCCGGCG<br>GGTGGGCCTCACACTTCACT
PLK3	NM_004073.4	CCCGATCGACTCCCTATCAG<br>CTCTCCTGGGCATGATTCTTAC
EGFR	NM_005228.5<br>(8 transcript variants)	TCTGGAAGTACGCAGACGCC<br>CGGGATCTTAGGCCCATTCGT

Transfection and Transduction:

Plasmid and siRNA transfections were carried out as described previously (Stilmann et al, 2009) using siRNA against *IKBKG* (GGAAGAGCCAACUGUGUGA), *IKBKB* (CUUAGAUACCUUCAUGAAA), or (139260 Silencer) (Invitrogen, USA).

Overexpression of p65 pECE 1-530 and p65 mutants was performed as described previously (Hatada EN, 1993).

Macrophage migration assay: THP1 cells were cultured for 24 h with 5 ng/ml Phorbol-12-myristat-13-acetat (PMA). Non-adherent cells were washed off and adherent macrophages were kept for additional 48 h under PMA-free conditions. Cells were trypsinized and seeded at $1x10^4$ cells/insert with an 8 µM pore-size in 24-well plates (Greiner). Supernatant from U2-OS pTRIPZ NFKBIA cells or RPMI control medium containing 1% FCS with and without 1 µg LPS was added to the lower chamber. Cells were allowed to migrate towards attractants for 16 h. The filter was rinsed with PBS, fixed in 4 % PFA and stained with 0.5 % crystal violet. Three fields of view were randomly selected and cell number was quantified by ImageJ software.

Chromatin immunoprecipitation (ChIP) assays: For ChIP, U2OS- cells were fixed using 2 mM disuccinimidylglutarate for 30 min followed by 1 % formaldehyde for 10 min at RT and lysed with 50 mM Tris-HCl, pH 8.0 / 5mM EDTA / 1 % SDS. Samples were then sonicated with the Bioruptor Plus (Diagenode; 12 cycles, intensity High, sonication 30 s/break 30 s per cycle). Chromatin was pre-cleared with BSA-saturated protein A-sepharose and incubated overnight at 4 °C with the p65 (Invitrogen PA1-186) (1,3 µg/$1x10^6$ cells) or P-(Ser468)-p65 (Cell signaling Technology #3039) (0.5µg/10^6 cells)specific antibody. Immuno-complexes were collected with BSA-saturated protein A-

sepharose for 1 h at 4 °C. The protein-DNA complexes were washed and then eluted using 1 % SDS / 0.1 M $NaHCO_3$. Reversion of the cross-linking, RNAse treatment, proteinase K digestion, and DNA purification by phenol-chloroform extraction were performed according to standard protocols.

Enrichment of genomic regions was determined by quantitative PCR (qPCR) using the CFX96 Real Time System (Bio-Rad) and GoTaq® qPCR Master Mix (Promega). ChIP-DNA was used corresponding to 3 x 10^5 cell equivalents with 4 ng of input DNA as control. All samples were analyzed in triplicates and values were normalized to non-recruiting intergenic regions on chromosome 4 and 12. The normalized enrichment (ΔΔCq) was calculated by the CFX manager software (Bio-Rad).

ChIP Primer sequences:

Gene	Acc.Nr.	sequence (5' → 3')	Primer Position (TSS=1)	Efficiency (%)
Reference	GRCh37:4:58100351-58100438	TGCAGGGGCAAGCATATTCA<br>GGAGATAAAGCTGGGCGACA	na	94,8
Reference	GRCh37:12:38676107-38676214	TGCCTTAGTTTTGTTGGTCCT<br>GTTGGGAGAAAAATGCACGGTTA	na	101,3
NFKBIA	GRCh37: 14:34940475-34943703:-1	TGCCAGGAACACTCAGCTCAT<br>GGCCGGGGTTTCTGGAG	792<br>861	94,1

Immunoprecipitation: Briefly, cells were harvested in ice-cold CHAPS buffer (TRIS-HCL pH7.4, 110 mM NaCl, and 50mM EDTA, supplemented with 1% CHAPS), incubated with primary antibodies overnight and one hour with G-Fast Flow Sepharose Beads (GE Healthcare).

Immunofluorescence staining was performed with the following antibodies: p65 (C20 Santa Cruz, USA); γH2AX (Millipore), and IκBα (C21 Santa Cruz, USA), IL-6 (Abcam), and p53 pSer15 (Cell Signaling). For confocal microscopy, in brief, ventral skin was fixed in 4% PFA overnight and incubated in 70% ethanol for 72 hours. Tissue was dehydrated by serial ethanol washes and embedded in paraffin. Sections were prepared in 5-micrometer thickness. After rehydration with serial dilution of ethanol, followed by demasking of the epitopes and blocking in donkey serum and TBST, samples were incubated in primary antibody overnight. Incubation with secondary antibody followed by counterstain with DAPI and fixation with immomount. Confocal microscopy was performed using LSM700 (Zeiss) Axio Imager M2. Lenses: 10x, 20x, 40x, 60x Zeiss Plan-Neofluoar and Plan-Apochromat. Processing: Zeiss Blue.

Senescence Associated-beta gal staining: SA-beta gal(Debacq-Chainiaux et al, 2009) performed in triplicates and 200 cells per field were counted per time point.

RNA-Seq data analysis: Read quality control was performed using Fastqc, Read trimming by quality and adapters using Trimmomatic, Read mapping using STAR without gene model annotations. For Read counting, Subread on protein coding genes from ENSEMBL version 70 - (strand aware counting) was used. For DE analysis,DESeq2 v1.10.1 was used.

Gene Ontology (GO Terms) analysis: RNA-Seq data were analysed using David Functional Annotation Microarray analysis https://david.ncifcrf.gov/ for enrichment of GO terms and annotations NF-κB targets genes were obtained from http://www.bu.edu/NF-κB/gene-resources/target-genes/.

Targets showing significant upregulation (P<0.05 and LOG2 > 0.5) were used for Gene Ontology Analysis (GO Term_ BP_ Fat); Thresholds: count 2; EASE 0.1. P Value: Benjamini.

Gene set enrichment analysis (GSE 139251) was performed as described (Subramanian et al. 2005) using the Molecular Signature Database v7.2.

LEGENDS TO SUPPLEMENTAL FIGURES

Figure S1. Two phases of NF-κB activation in human diploid fibroblasts and cancer cell lines in response to DNA damage

A) mRNA from U2-OS cells analyzed by RT-qPCR. Cells were irradiated (20 Gy) once and RNA harvested at indicated time points post a single dose irradiation. h= hours, D = days, ut = untreated. Expression of CDKN1A was normalized to two control reference genes. Statistical significance from n = 3 replicates was determined by ANOVA with Tukey multiple comparisons test. SD *** = p < 0.001.

(B) Time course analysis of U2-OS cells either untreated or irradiated (20 Gy). Duplications counted at indicated time points. Statistical significance from n = 3 replicates was determined by ANOVA with Tukey multiple comparisons test. SD *** = p < 0.001 for time point day 5, not significant for day 2.

(C) The proliferation rate of irradiated (20 Gy IR 7 days prior to analysis) and non-irradiated U2-OS cells was evaluated according to BrdU uptake by flow cytometry analysis in n = 4 independent experiments. U2-OS cells were cultured with or without BrdU for 12 hours. A representative histogram shows BrdU staining for irradiated with 20 Gy (green line), non-irradiated (red line) and BrdU-untreated (blue line) cells.

(D) Immunofluorescence microscopy of U2-OS cells stained with p65 antibody (green), γH2AX (red), or DAPI (blue). Cells were irradiated once (20 Gy) at indicated time points prior to staining. h= hours, D = days, ut = untreated. Nuclear γH2AX foci served as indication of DNA damage. DAPI staining served to visualize nuclei. Representative images are shown from n = 3 replicate experiments. Scale bar: 25 μm.

Right panels: Quantitation from n = 3 experiments with antibodies shown. Nuclear localization was calculated as percentage. Statistical significance was determined by ANOVA with Tukey multiple comparisons test. SD *** = $p < 0.001$.

(E) SDS-PAGE western blot on nuclear lysates of U2-OS cells irradiated with 20 Gy at time points shown prior to harvest. PARP1 represents loading control. Representative gel shown from n = 3 experiments.

(F) SDS-PAGE western blot on nuclear lysates of HD WI-38 cells at indicated time points after IR (40 Gy). Representative images from n = 3 experiments.

(G) as in (F) with BJ cells from n = 3 experiments.

(H) RT-qPCR performed on skin from irradiated (5 Gy) n = 6 mice (female) for each condition, at time points indicated post irradiation. Statistical significance was determined by ANOVA with Tukey multiple comparisons test. SD * = $p < 0.05$, SD ** = $p < 0.01$.

(I) Kidney cells from IκBα$^{\Delta N}$ mice (ΔN, n = 4) or control littermates (WT, n = 4) were irradiated (40 Gy) either 1.5h or seven days prior to harvest or left untreated (ut). RNA was analyzed by qRT-PCR for indicated genes normalized to two references. Statistical significance was determined by ANOVA with Tukey multiple comparisons test. SD * = $p < 0.05$, SD ** = $p < 0.01$.

Figure S2: Loss of IκBα in senescence leads to two phases of NF-κB activation

(A) Whole cell fractions of BJ cells irradiated with 40 Gy cells analyzed by SDS-PAGE western blot at time points after IR as indicated. Representative image from n = 3 experiments.

(B) as in (A) with WI-38 cells. Representative image from n = 3 experiments.

(C) RT-qPCR performed on RNA from cell lines indicated, which were untreated or analyzed 1.5 hours or 5 days after irradiation (20 Gy), as indicated. *NFKBIA* mRNA levels shown are relative to two reference controls. Statistical significance was determined from n = 3 replicates by ANOVA with Tukey multiple comparisons test. SD ** = $p < 0.01$, *** = $p < 0.001$.

(D) RASV12-ER TIG3 cells were treated with tamoxifen 100 nM for 6 hours, for 2 days or for 5 days or left untreated. Whole cell lysates were analyzed by SDS-PAGE western blot for IL-8 and actin as loading control. Lower panel, samples analyzed by EMSA with an H2K NF-κB binding site probe. Representative of n = 3 experiments.

(E) RASV12-ER TIG3 cells were treated with tamoxifen. 100nM and analyzed by RT-qPCR after the indicated time points for mRNA as indicated. Statistical significance was determined from n = 3 replicates by ANOVA with Tukey multiple comparisons test. SD *** = $p < 0.001$. n.s. not significant.

(F) U2-OS cells bearing doxycycline (Dox) inducible shRNA against *NFKBIA* were analyzed by SDS-PAGE western blot after irradiation (20 Gy) at time points indicated. Dox+ cells were treated for the duration of the experiments with Dox to knockdown *NFKBIA.* Representative gel from n = 3 experiments is shown.

(G) RT-qPCR of U2-OS cells transiently transfected with a construct encoding IκBα S32AS36A or empty vector. Cells were transfected two days after irradiation (IR). Cells were irradiated with 20 Gy 6 days prior to harvest (IR) or left untreated. Statistical significance was determined from n = 3 replicates by ANOVA with Tukey multiple comparisons test. SD *** = $p < 0.001$.

(H) Human Cytokine Arrays assayed with cultured media from U2-OS cells that were either untreated, irradiated (20 Gy) for 6 days (second phase), or treated with Dox to

deplete IκBα (*NFKBIA* shRNA). Relative secretion of cytokines/chemokines was normalized to two references and calculated as a ratio to the untreated sample, which was set to 1. Statistical significance was determined from n = 2 replicates by ANOVA with Tukey multiple comparisons test. SD * = $P < 0.05$, ** = $p < 0.01$, *** = $p < 0.001$.

(I) Macrophage migration trans-well assay. Macrophages were exposed to U2-OS cell medium from wild type cells (neg. control), LPS treated cells (pos. control), untreated cells (U2-OS cells expressing IκBα shRNA not treated with Dox), senescent cells (cells irradiated (20 Gy) 5 days prior to medium collection) or from cells with IκBα depletion (U2-OS cells expressing IκBα shRNA plus Dox). Left panel: Migrated cells were stained with crystal violet. Representative images from n = 3 experiments. Right panel: Quantitation from the trans-well assay. Scale bar: 100 μm. Statistical significance was determined from n = 3 replicates by ANOVA with Tukey multiple comparisons test. SD *** = $p < 0.001$.

Figure S3: Constitutive NF-κB activation does not trigger senescence associated proliferative arrest

(A) Cell duplication was measured at indicated time points in biological triplicates in non-irradiated cells bearing shRNA against *NFKBIA*, treated with Dox to induce knockdown (shRNA *NFKBIA*) or with water (untreated) No significant difference in duplication rate was observed at day 6 (Student *t* test, from n = 3).

(B) Analysis performed as in S1C. The proliferation rate of irradiated (20 Gy, 7 days prior to analysis) and non-irradiated U2-OS cells bearing inducible shRNA against *NFKBIA*, as described above, was evaluated according to BrdU uptake by flow cytometry analysis. Cells were cultured with or without BrdU for 12 hours. A representative

histogram shows BrdU staining for irradiated Dox treated cells (green line), irradiated Dox untreated cells (orange line), non-irradiated Dox untreated cells (red line) and non-irradiated Dox treated cells (blue line).

(C) GSEA was performed comparing expression data from small intestine of *Nfkbia* KO mice versus wild type (GSE 139251) with 35 gene sets from the Molecular Signature Database v7.2. containing the term ”senescence”. Enrichment plot for the gene set REACTOME_CELLULAR_SENESCENCE is shown. NES, Normalized Enrichment Score; FDR, False Discovery Rate.

(D) Paraffin sections from small intestines from untreated or irradiated (5 Gy) C57BL/5 mice (female), or from untreated *NFKBIA* mice (male and female) stained with p53 pSer15 antibody and DAPI to detect DNA damage. Representative sections shown from n = 6 mice from each group. Scale bar: 50 µm. Upper right corner: zoom in to show nuclear p53 pSer15 foci.

(E) Quantitation from (D) showing percentage per intestinal crypt of cells with nuclear p53 pSer15 foci. Statistical significance was determined from n = 6 replicates by ANOVA with Tukey multiple comparisons test. SD ** = $p < 0.01$, *** = $p < 0.001$.

Figure S4. Loss of p65 does not rescue cells from proliferative arrest

(A) SDS-PAGE western blot from U2-OS cells expressing Dox inducible shRNA against *RELA*. Cells were treated with Dox to induce knockdown and irradiated with 20 Gy at time points shown prior to harvest. Representative image shown from n = 2 experiments.

(B) RT-qPCR of U2-OS cells (as in A), showing levels of p16 (CDKN2A). From n = 3 replicates. Statistical significance was determined by ANOVA with Tukey multiple comparisons test. SD ** = $p < 0.01$, *** = $p < 0.001$.

(C) Analysis performed as in S1C. The proliferation rate of irradiated (20 Gy, 7 days prior to analysis) and non-irradiated U2-OS cells bearing inducible shRNA against *RELA*, as described above, was evaluated according to BrdU uptake by flow cytometry analysis. Cells were cultured with or without BrdU for 12 hours. A representative histogram from n = 4 experiments shows BrdU staining for irradiated Dox treated cells (green line), irradiated Dox untreated cells (orange line), non-irradiated Dox untreated cells (red line) and non-irradiated Dox treated cells (blue line).

Figure S5. IKK- independent activation of p65 in senescence

(A) SDS-PAGE western blot from U2-OS cells irradiated with 20 Gy and harvested at indicated time points. Knockdown of ATM (or control with scrambled siRNA) was performed two days prior to harvest. Representative image shown from n = 3 experiments.

(B) as in (A) showing RT-qPCR of p65 target genes from n = 3 replicates. Statistical significance was determined by ANOVA with Tukey multiple comparisons test. SD ** = $p < 0.01$, *** = $p < 0.001$. n.s. not significant.

(C) siRNAs against *IKBKG* or scrambled control were transfected into U2-OS cells three days prior to harvest. Nuclear translocation of p65 was examined in the nuclear lysates of U2-OS cells at 1.5 hours or 5 days post irradiation by SDS-PAGE western blot, as indicated. Cytoplasmic lysates were used to show knockdown efficiency. Representative

gel from n = 3 experiments. PARP1 serves as loading and fractionation control for the nuclear fraction.

(D) Heatmap showing IKKβ – independent, p65-dependent transcripts, analyzed by RT-qPCR following siRNA knockdown of CHUK (IKKα) versus scrambled control (Scr). Expression normalized to two reference controls. n = 3 biological replicates. Statistical significance was determined by ANOVA with Tukey multiple comparisons test, available in source data table.

SUPPLEMENTAL REFERENCES

Debacq-Chainiaux F, Erusalimsky JD, Campisi J, Toussaint O (2009) Protocols to detect senescence-associated beta-galactosidase (SA-betagal) activity, a biomarker of senescent cells in culture and in vivo. *Nature protocols* **4:** 1798-1806

Hatada EN NM, Scheidereit C. (1993) Common structural constituents confer I kappa B activity to NF-kappa B p105 and I kappa B/MAD-3. *The EMBO journal* **12:** 2781-2788

Rehm A, Gatjen M, Gerlach K, Scholz F, Mensen A, Gloger M, Heinig K, Lamprecht B, Mathas S, Begay V, Leutz A, Lipp M, Dorken B, Hopken UE (2014) Dendritic cell-mediated survival signals in Emu-Myc B-cell lymphoma depend on the transcription factor C/EBPbeta. *Nature communications* **5:** 5057

Stilmann M, Hinz M, Arslan SC, Zimmer A, Schreiber V, Scheidereit C (2009) A nuclear poly(ADP-ribose)-dependent signalosome confers DNA damage-induced IkappaB kinase activation. *Molecular cell* **36:** 365-378

Subramanian A, Tamayo P, Mootha VK, Mukherjee S, Ebert BL, Gillette MA, et al. Gene set enrichment analysis: a knowledge-based approach for interpreting genome-wide expression profiles. Proceedings of the National Academy of Sciences of the United States of America. 2005;102(43):15545-50. Epub 2005/10/04.

Figure S1

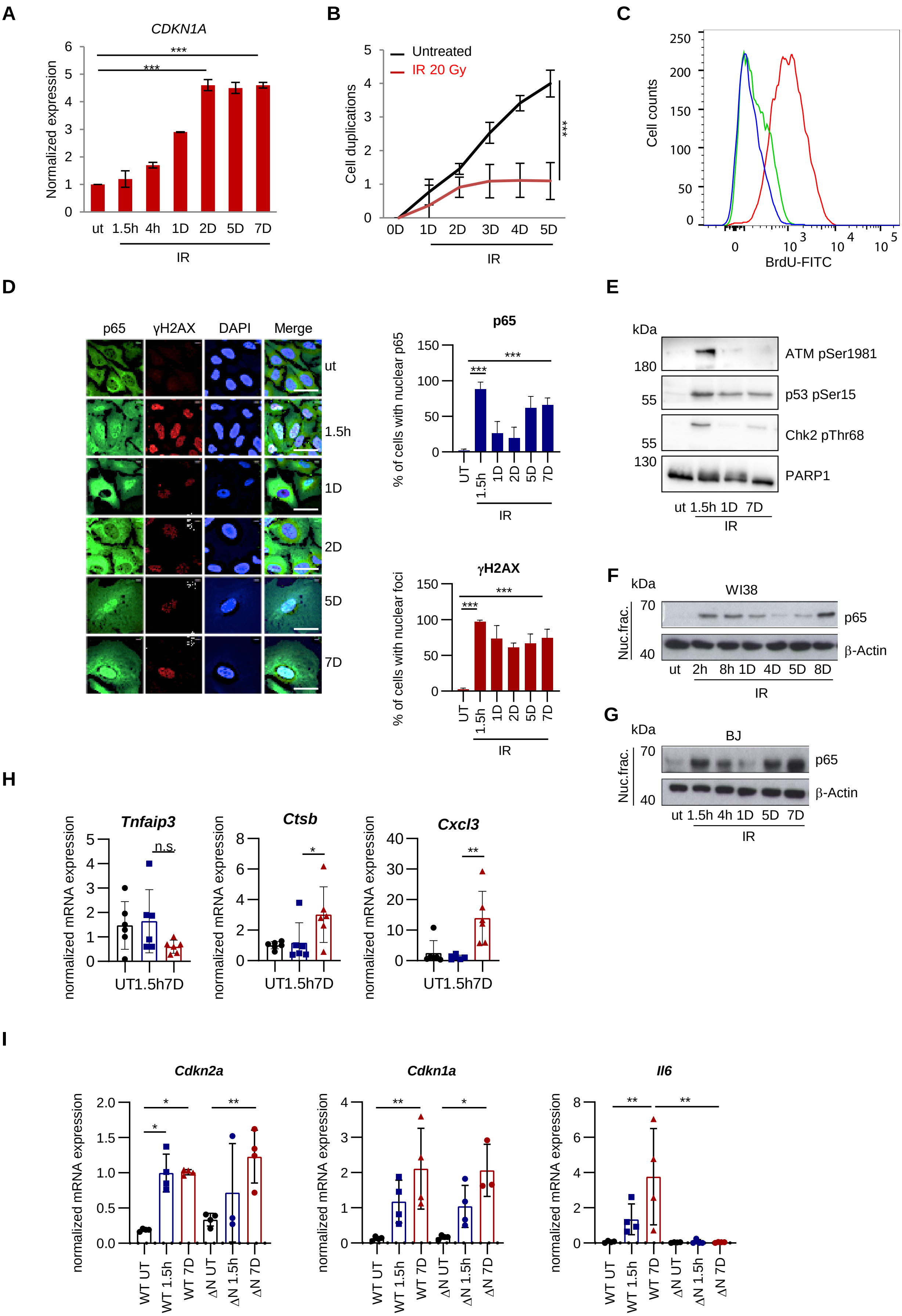

A
CDKN1A
Normalized expression
ut 1.5h 4h 1D 2D 5D 7D
IR
B
Untreated
IR 20 Gy
Cell duplications
0D 1D 2D 3D 4D 5D
IR
C
Cell counts
BrdU-FITC
D
p65 γH2AX DAPI Merge
ut
1.5h
1D
2D
5D
7D
p65
% of cells with nuclear p65
UT 1.5h 1D 2D 5D 7D
IR
γH2AX
% of cells with nuclear foci
UT 1.5h 1D 2D 5D 7D
IR
E
kDa
ATM pSer1981
p53 pSer15
Chk2 pThr68
PARP1
ut 1.5h 1D 7D
IR
F
WI38
Nuc.frac.
p65
β-Actin
ut 2h 8h 1D 4D 5D 8D
IR
G
BJ
Nuc.frac.
p65
β-Actin
ut 1.5h 4h 1D 5D 7D
IR
H
Tnfaip3
Ctsb
Cxcl3
normalized mRNA expression
n.s.
UT1.5h7D
I
Cdkn2a
Cdkn1a
Il6
normalized mRNA expression
WT UT WT 1.5h WT 7D ΔN UT ΔN 1.5h ΔN 7D

Figure S2

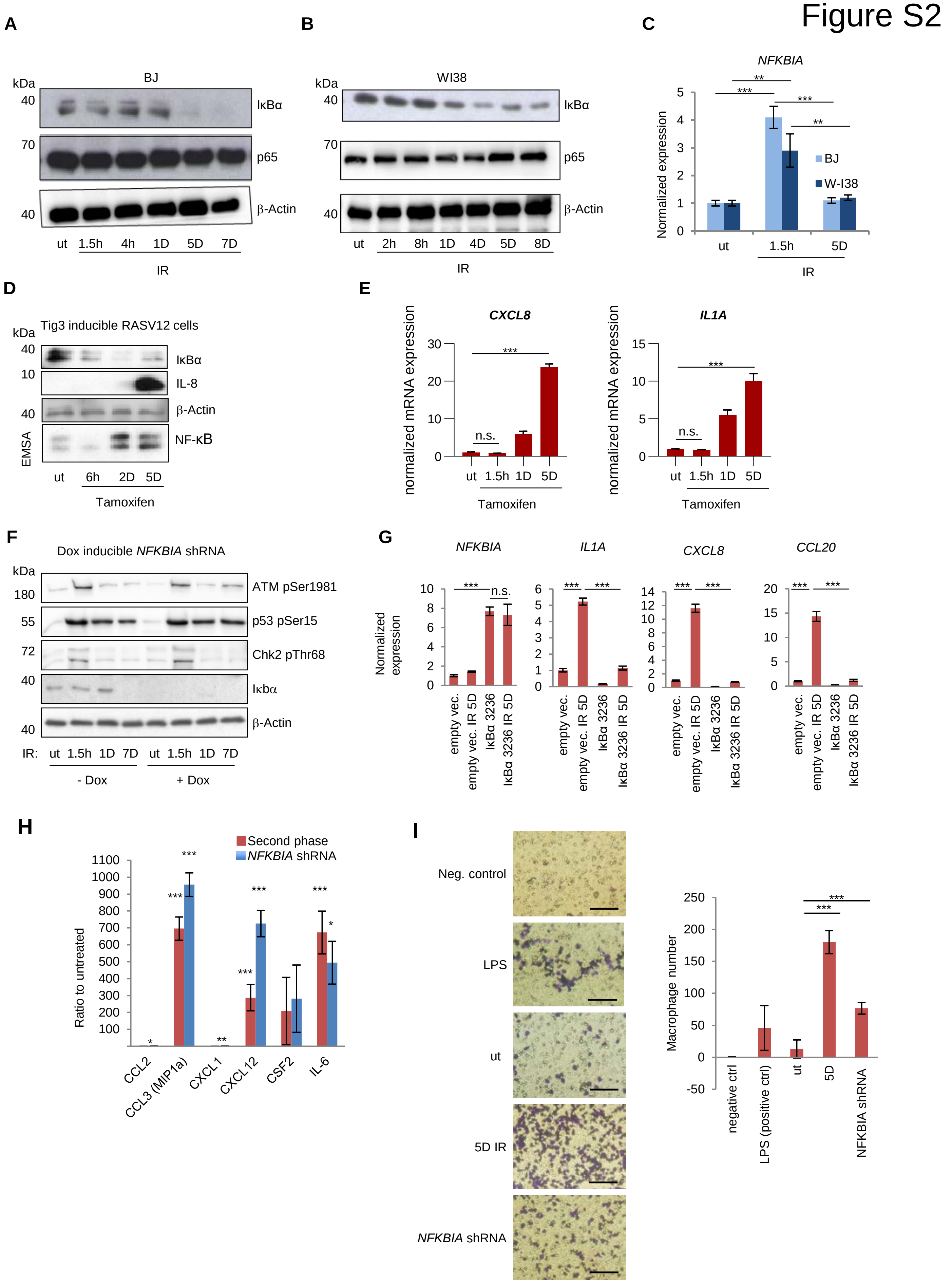
A
B
C
BJ
kDa
40
IκBα
70
p65
40
β-Actin
ut 1.5h 4h 1D 5D 7D
IR
WI38
kDa
40
IκBα
70
p65
40
β-Actin
ut 2h 8h 1D 4D 5D 8D
IR
NFKBIA
Normalized expression
0 1 2 3 4 5
**
***
***
**
BJ
W-I38
ut 1.5h 5D
IR
D
E
Tig3 inducible RASV12 cells
kDa
40
IκBα
10
IL-8
40
β-Actin
EMSA
NF-κB
ut 6h 2D 5D
Tamoxifen
CXCL8
normalized mRNA expression
0 10 20 30
***
n.s.
ut 1.5h 1D 5D
Tamoxifen
IL1A
normalized mRNA expression
0 5 10 15
***
n.s.
ut 1.5h 1D 5D
Tamoxifen
F
Dox inducible NFKBIA shRNA
kDa
180
ATM pSer1981
55
p53 pSer15
72
Chk2 pThr68
40
Iκbα
40
β-Actin
IR: ut 1.5h 1D 7D ut 1.5h 1D 7D
- Dox
+ Dox
G
Normalized expression
NFKBIA
0 2 4 6 8 10
***
n.s.
IL1A
0 1 2 3 4 5 6
*** ***
CXCL8
0 2 4 6 8 10 12 14
*** ***
CCL20
0 5 10 15 20
*** ***
empty vec.
empty vec. IR 5D
IκBα 3236
IκBα 3236 IR 5D
H
Second phase
NFKBIA shRNA
Ratio to untreated
100 200 300 400 500 600 700 800 900 1000 1100
*
***
***
**
***
***
***
*
CCL2
CCL3 (MIP1a)
CXCL1
CXCL12
CSF2
IL-6
I
Neg. control
LPS
ut
5D IR
NFKBIA shRNA
Macrophage number
-50 0 50 100 150 200 250
***
***
negative ctrl
LPS (positive ctrl)
ut
5D
NFKBIA shRNA

Figure S3

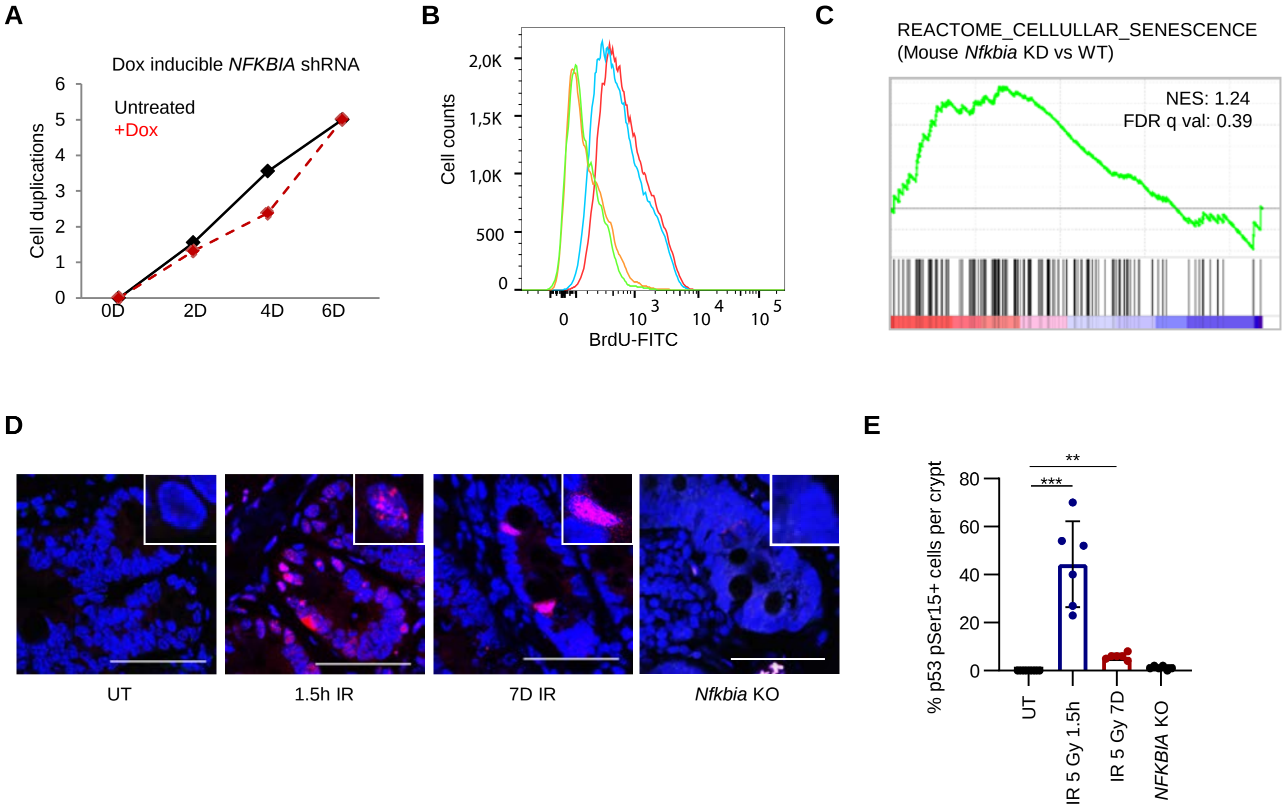
A
Dox inducible NFKBIA shRNA
Untreated
+Dox
Cell duplications
0
1
2
3
4
5
6
0D
2D
4D
6D
B
Cell counts
2,0K
1,5K
1,0K
500
0
0
10 3
10 4
10 5
BrdU-FITC
C
REACTOME_CELLULLAR_SENESCENCE
(Mouse Nfkbia KD vs WT)
NES: 1.24
FDR q val: 0.39
D
UT
1.5h IR
7D IR
Nfkbia KO
E
% p53 pSer15+ cells per crypt
0
20
40
60
80
**
***
UT
IR 5 Gy 1.5h
IR 5 Gy 7D
NFKBIA KO

Figure S4

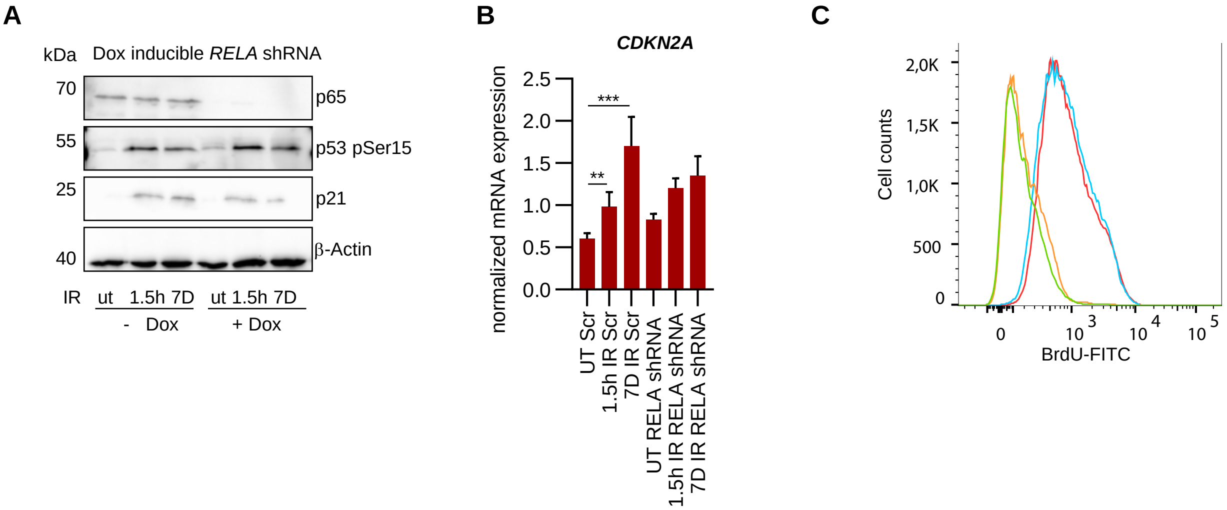
A
kDa
Dox inducible *RELA* shRNA
70
55
25
40
p65
p53 pSer15
p21
β-Actin
IR ut 1.5h 7D ut 1.5h 7D
- Dox
+ Dox
B
CDKN2A
normalized mRNA expression
2.5
2.0
1.5
1.0
0.5
0.0
***
**
UT Scr
1.5h IR Scr
7D IR Scr
UT RELA shRNA
1.5h IR RELA shRNA
7D IR RELA shRNA
C
Cell counts
2,0K
1,5K
1,0K
500
0
0
10 3
10 4
10 5
BrdU-FITC

Figure S5

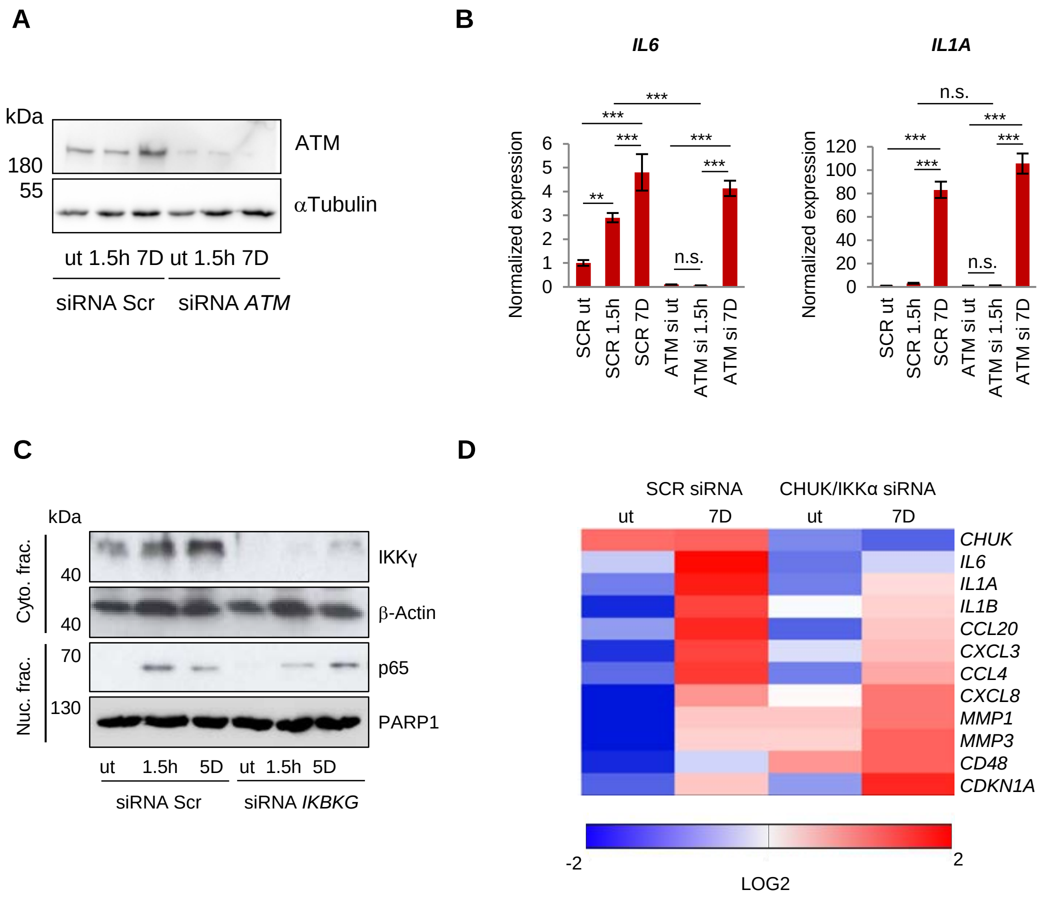
A
kDa
180
55
ATM
αTubulin
ut 1.5h 7D ut 1.5h 7D
siRNA Scr
siRNA ATM
B
IL6
IL1A
Normalized expression
SCR ut
SCR 1.5h
SCR 7D
ATM si ut
ATM si 1.5h
ATM si 7D
n.s.
C
kDa
Cyto. frac.
Nuc. frac.
IKKγ
β-Actin
p65
PARP1
ut 1.5h 5D ut 1.5h 5D
siRNA Scr
siRNA IKBKG
D
SCR siRNA
CHUK/IKKα siRNA
ut
7D
CHUK
IL6
IL1A
IL1B
CCL20
CXCL3
CCL4
CXCL8
MMP1
MMP3
CD48
CDKN1A
-2
2
LOG2
