# Supplementary material for: Transcriptional repression of NFKBIA triggers constitutive IKK‐ and proteasome‐independent p65/RelA activation in senescence
Source: EMBO J. 2021 Jan 18;40(6):e104296. doi: 10.15252/embj.2019104296 (PMC7957429; doi:10.15252/embj.2019104296)
Supplement: Supplementary file 6 — Source Data for Figure 1 [file EMBJ-40-e104296-s008.pdf]

OS 1B

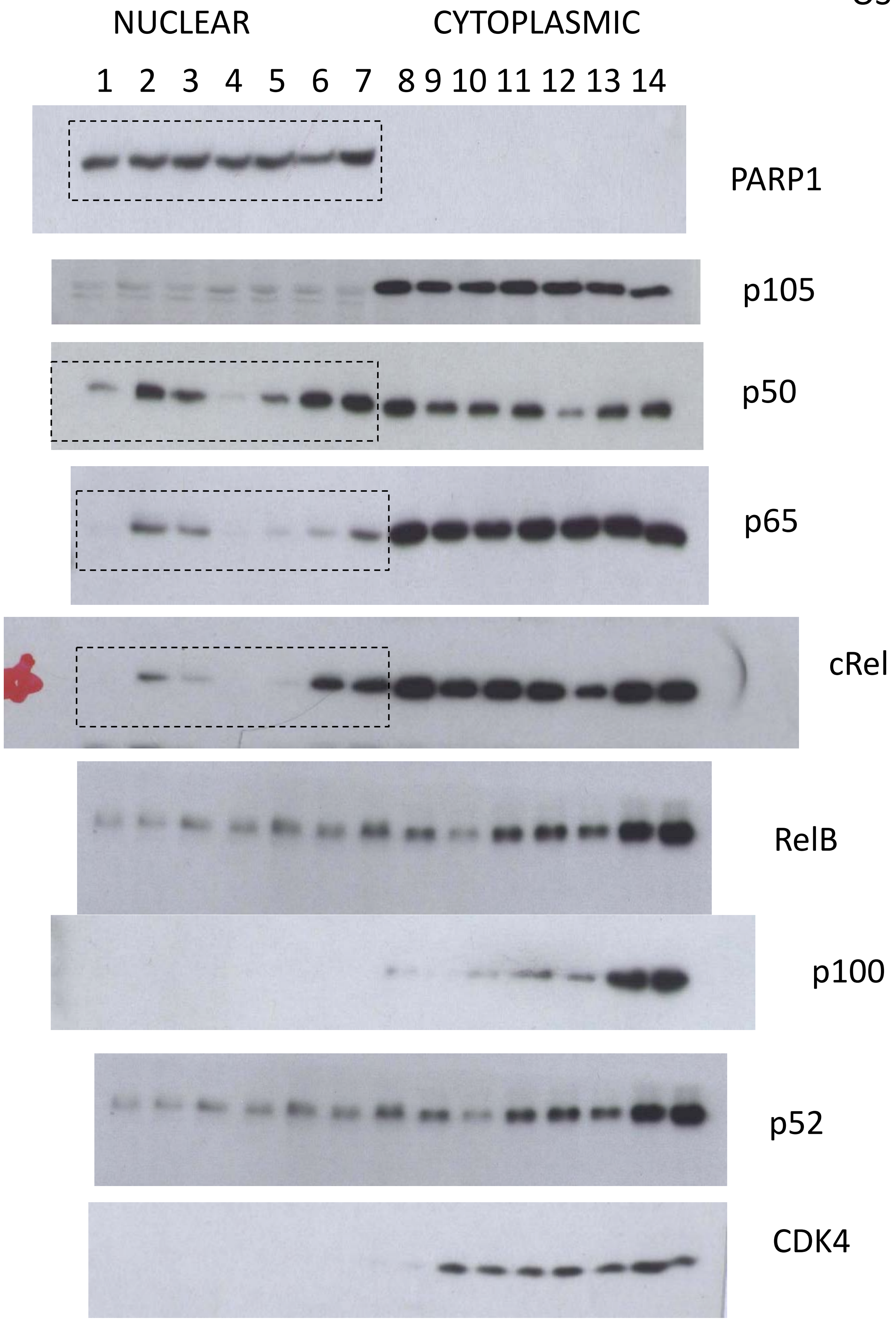

Uncropped blots used for Fig 1B. Also shown loading and fractionation controls (CDK4 for cytoplasmic, and PARP1 for nuclear). Non-canonical subunits are also shown as well as precursors (p105 (for p50 and p100 for p52).
