# Supplementary figures and images for: Transcriptional repression of NFKBIA triggers constitutive IKK‐ and proteasome‐independent p65/RelA activation in senescence
Source: EMBO J. 2021 Jan 18;40(6):e104296. doi: 10.15252/embj.2019104296 (PMC7957429; doi:10.15252/embj.2019104296)

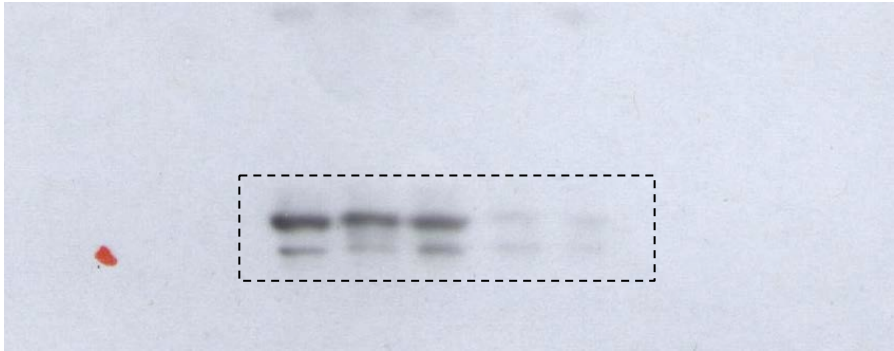

Ikappa B alpha

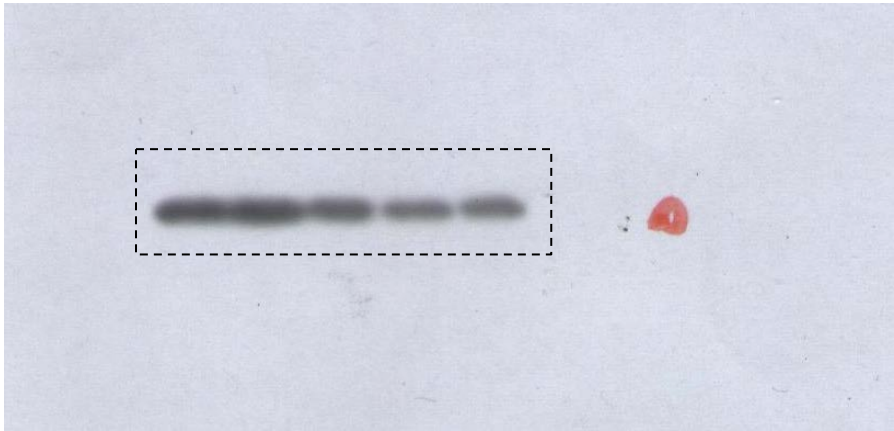

Actin

Uncropped blots for Fig 2A.

Supplement: Supplementary file 7 — Source Data for Figure 2 [file EMBJ-40-e104296-s010.zip › Figure 2 SD.pdf]

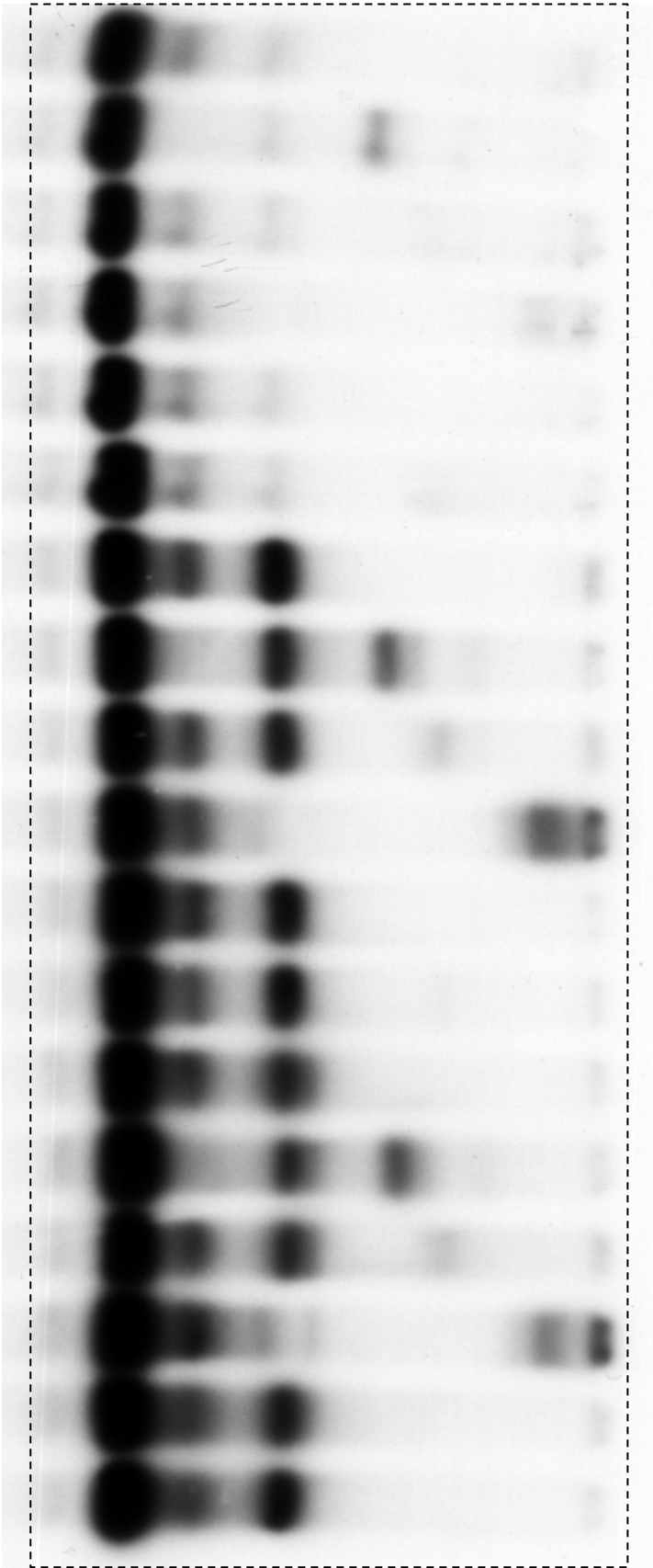

Uncropped X-ray film used for Fig 3A.

Supplement: Supplementary file 8 — Source Data for Figure 3 [file EMBJ-40-e104296-s003.zip › Figure 3 SD.pdf]
