# Supplementary material for: Transcriptional repression of NFKBIA triggers constitutive IKK‐ and proteasome‐independent p65/RelA activation in senescence
Source: EMBO J. 2021 Jan 18;40(6):e104296. doi: 10.15252/embj.2019104296 (PMC7957429; doi:10.15252/embj.2019104296)
Supplement: Supplementary file 9 — Source Data for Figure 4 [file EMBJ-40-e104296-s006.zip › Figure 4 SD.pdf]

OS 4A

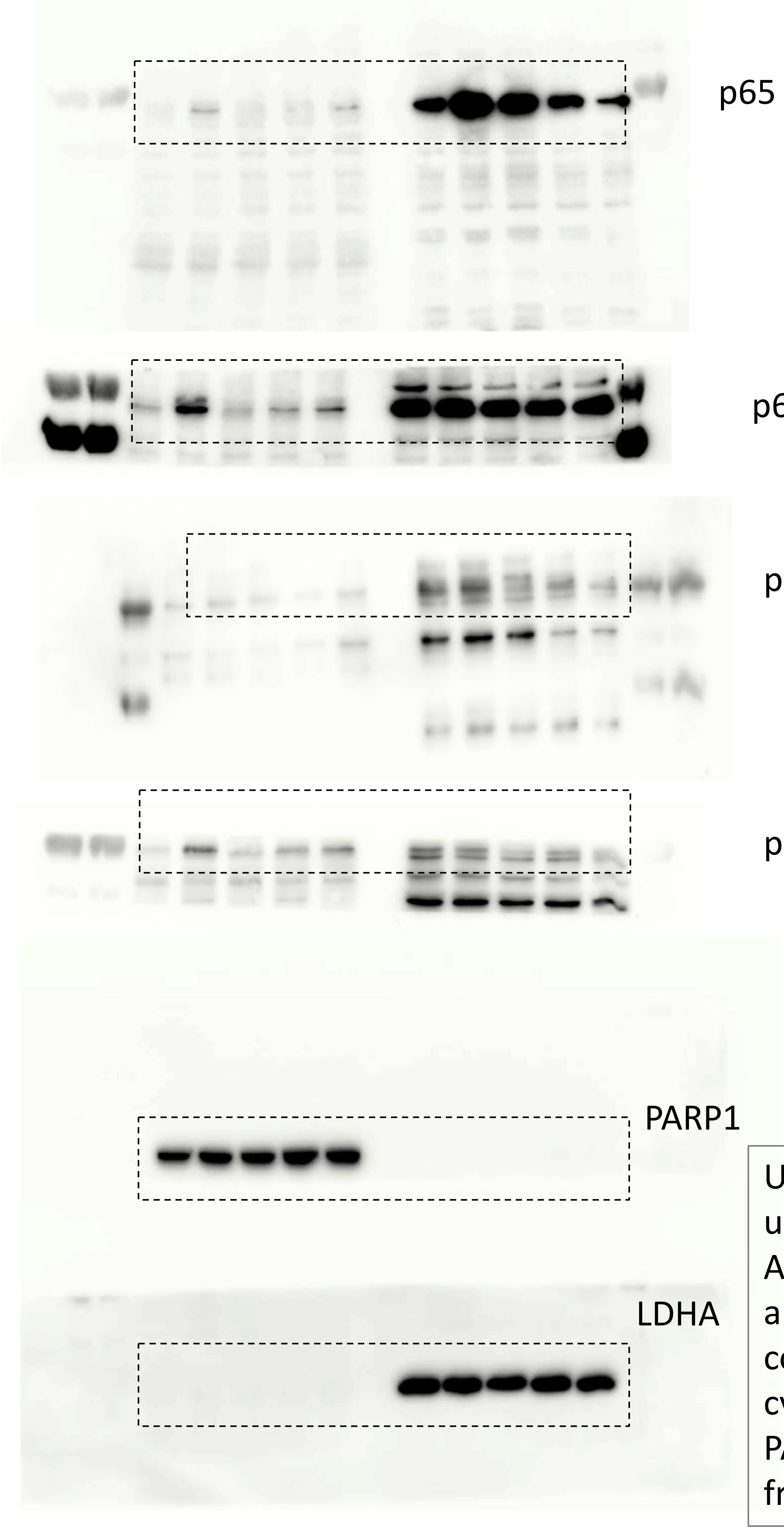

Uncropped blots used for Fig 4A. Also shown loading and fractionation controls (LDHA for cytoplasmic, and PARP1 for nuclear fraction).

OS 4B

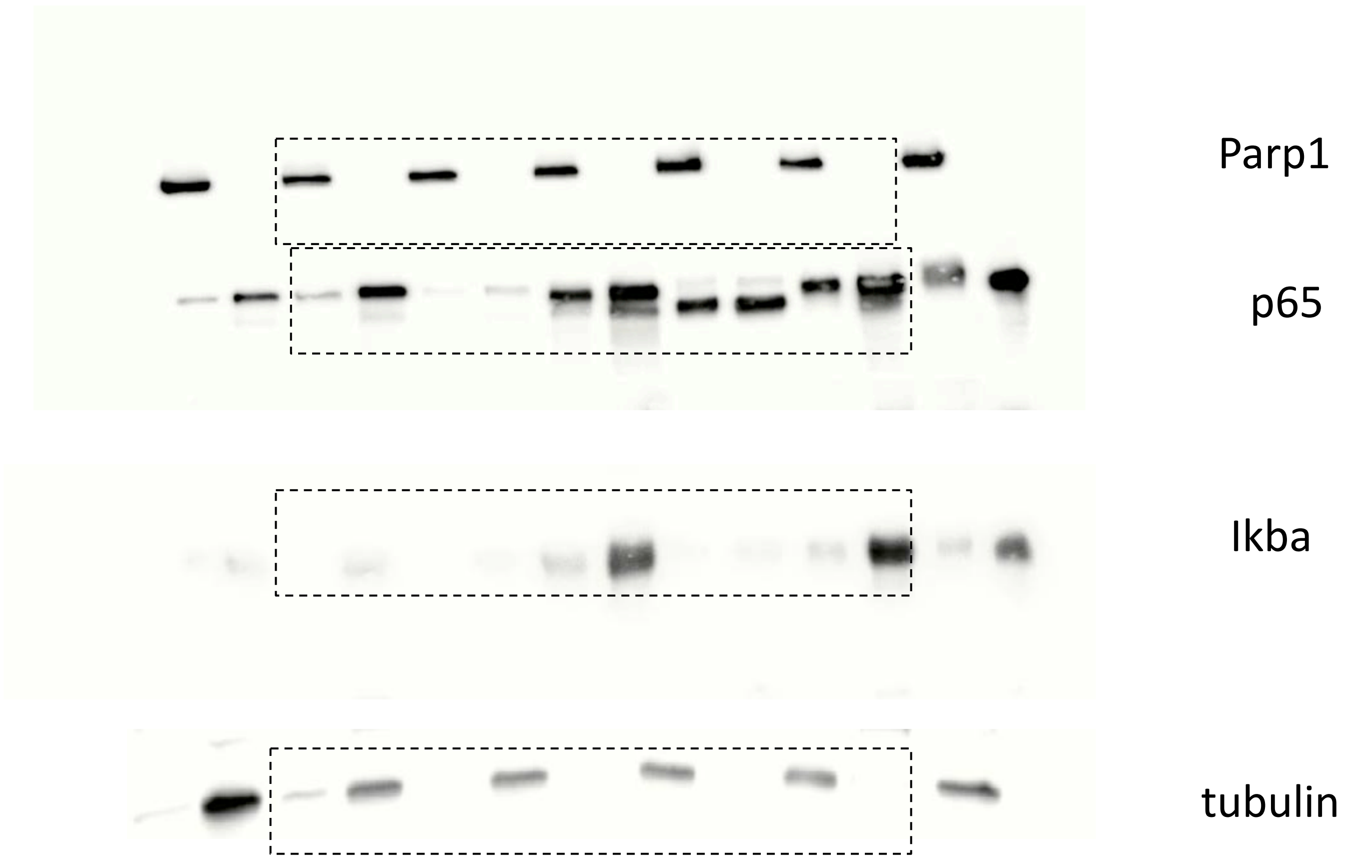

Uncropped blots used for Fig 4B. Also shown loading and fractionation controls (tubulin for cytoplasmic, and Parp1 for nuclear fraction).

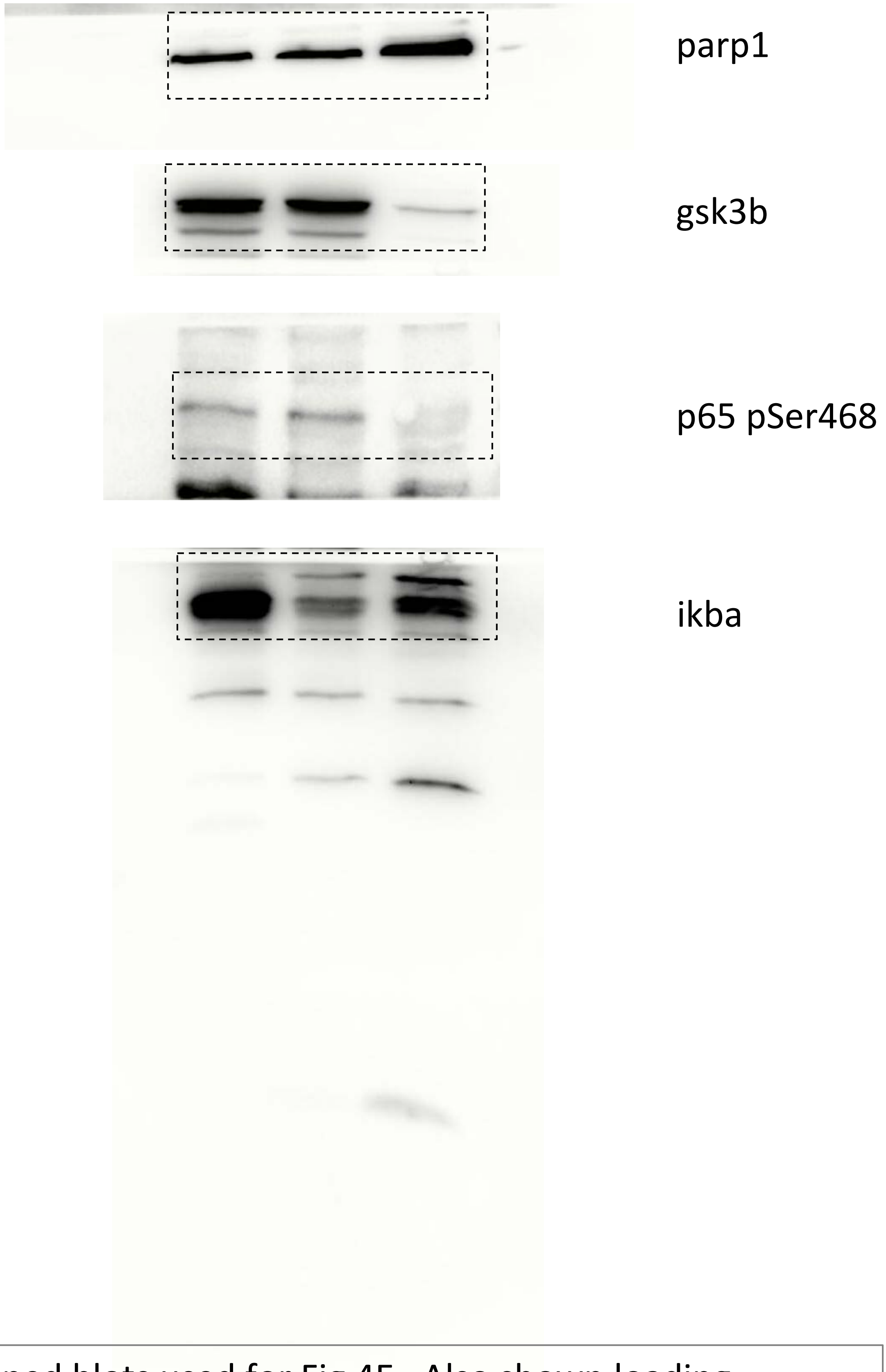

Uncropped blots used for Fig 4E. Also shown loading and fractionation control (Parp1 for nuclear fraction).

OS 4F

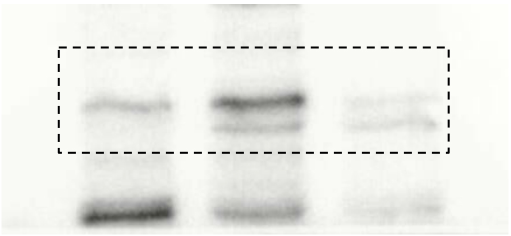

p65 pSer468

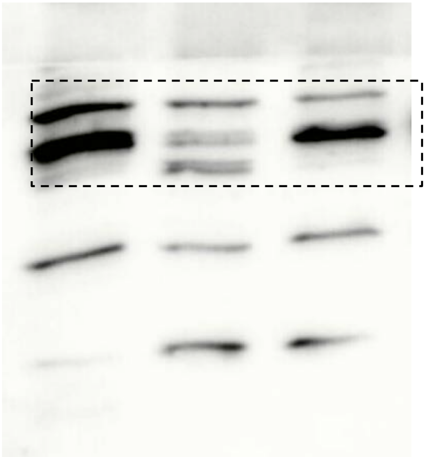

IκBα

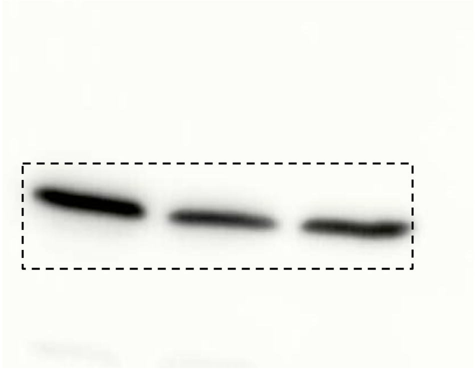

LDHA

Uncropped blots used for Fig 4F. Also shown loading control LDHA.
