# Supplementary material for: Transcriptional repression of NFKBIA triggers constitutive IKK‐ and proteasome‐independent p65/RelA activation in senescence
Source: EMBO J. 2021 Jan 18;40(6):e104296. doi: 10.15252/embj.2019104296 (PMC7957429; doi:10.15252/embj.2019104296)
Supplement: Supplementary file 10 — Source Data for Figure 5 [file EMBJ-40-e104296-s002.pdf]

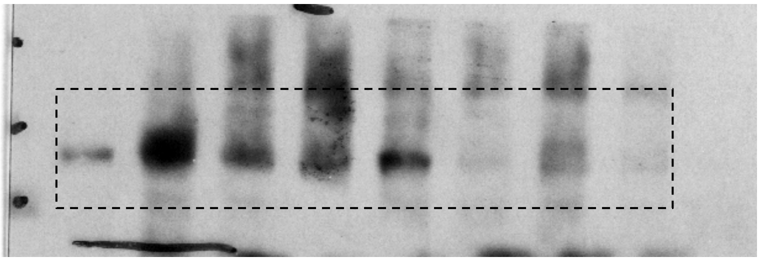

IKKα/β pSer 176/180

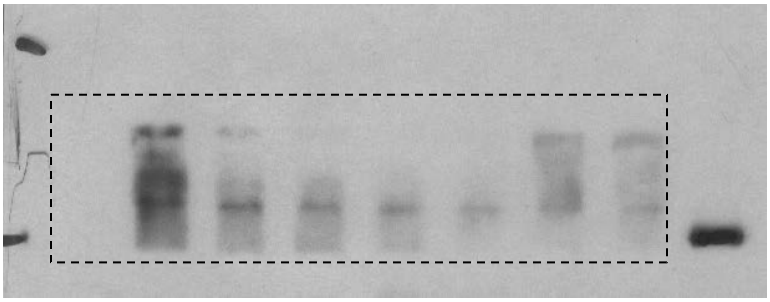

ATM pSer 1981

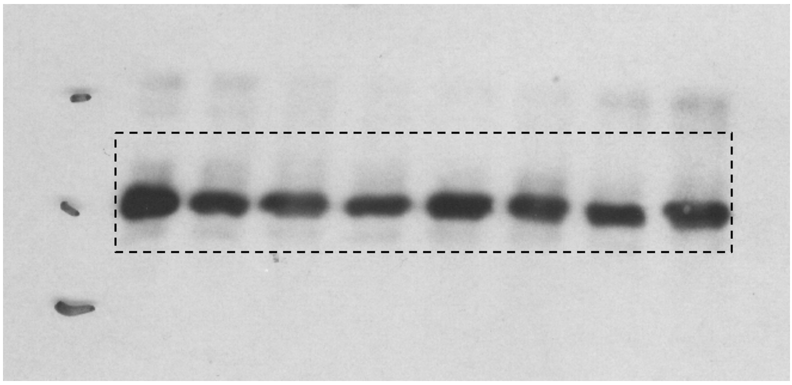

LDHA

Uncropped films used for Fig 5B. Also shown loading control LDHA.

OS 5C

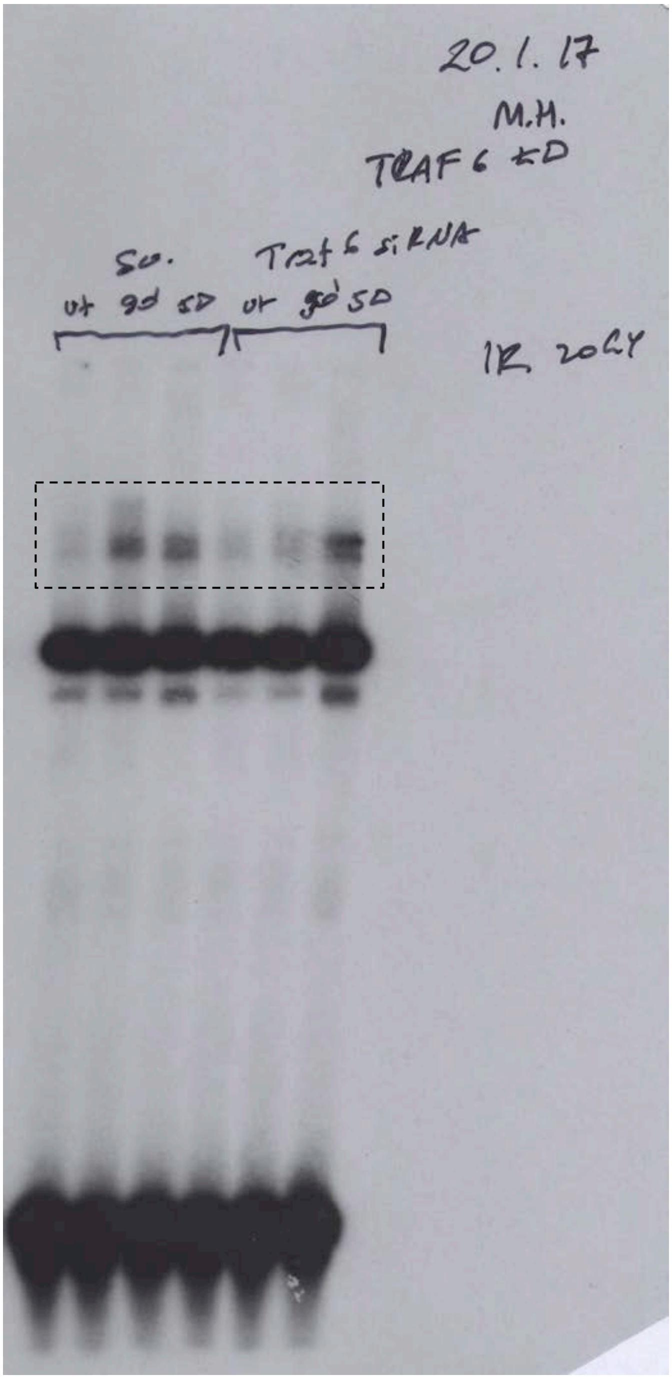

NF-kB EMSA

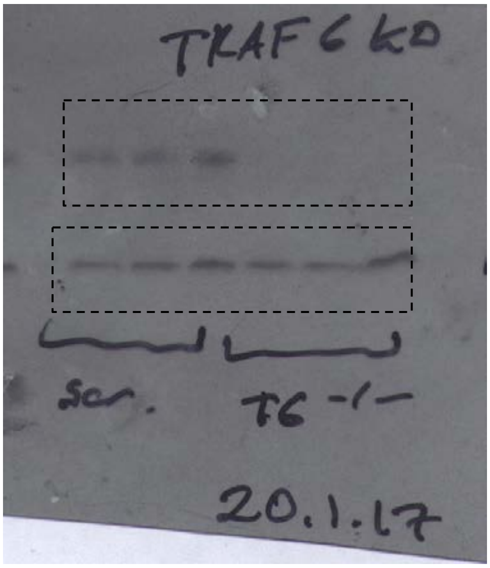

TRAF6-WB

Uncropped X-ray film (EMSA) and TRAF6 blot used for Fig 5C.

OS 5E

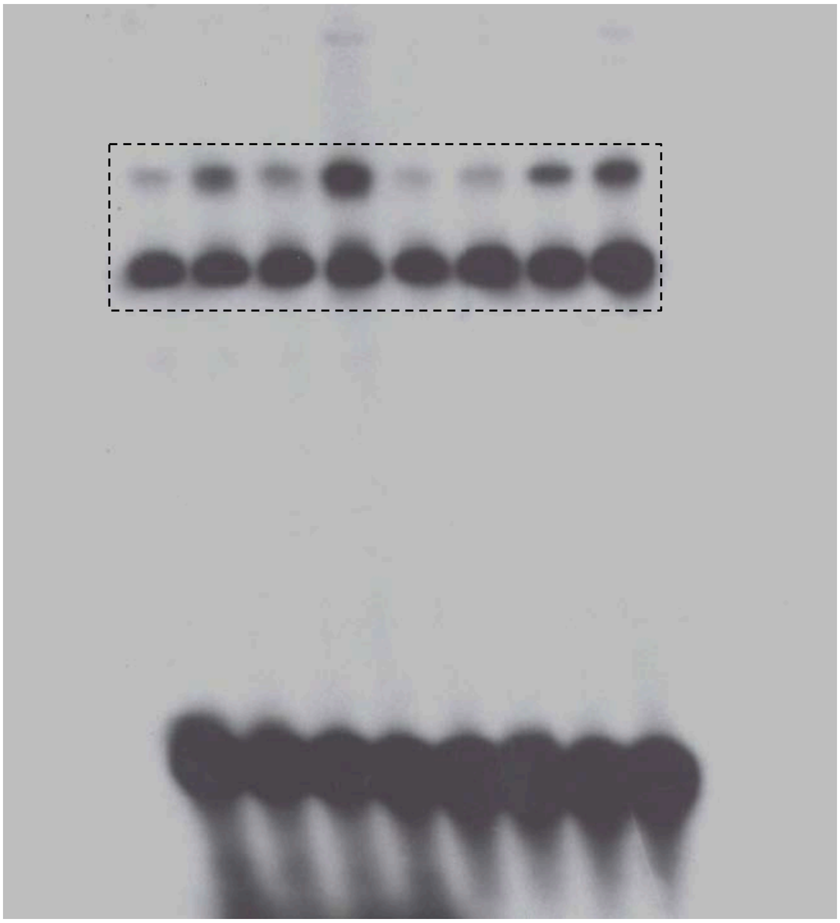

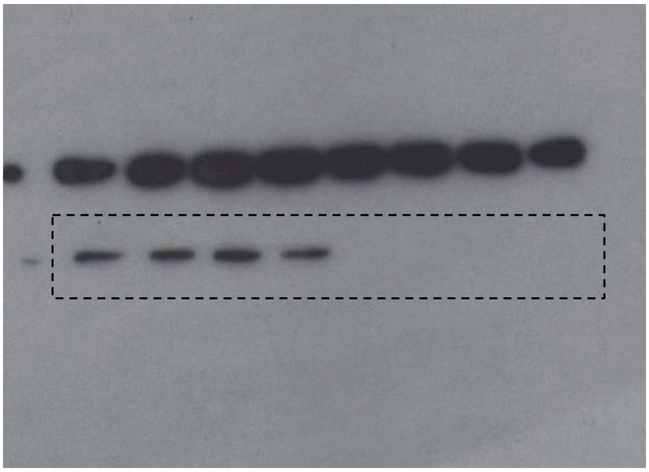

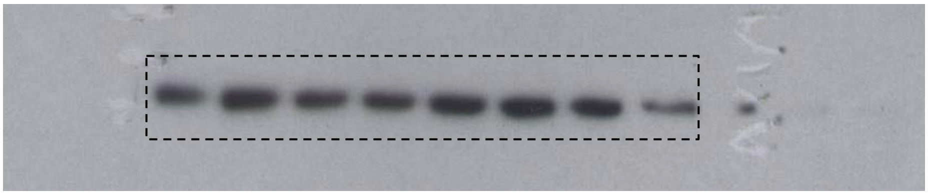

Uncropped X-ray film (EMSA) and blots used for Fig 5E. Also shown loading control β-Actin.
